# Inhibition of γ-secretase induces G2/M arrest and triggers apoptosis in renal cell carcinoma

**DOI:** 10.3892/ol.2014.2078

**Published:** 2014-04-22

**Authors:** KERONG WU, LI ZHANG, YIWEI LIN, KAI YANG, YUE CHENG

**Affiliations:** 1 Department of Urology, Ningbo First Hospital, School of Medicine, Ningbo University, Ningbo, Zhejiang 315000, P.R. China; 2 Department of Urology, Zhongshan Hospital, Shanghai Medical College, Fudan University, Shanghai 200000, P.R. China; 3 Department of Urology, First Affiliated Hospital, School of Medicine, Zhejiang University, Hangzhou, Zhejiang 310003, P.R. China

**Keywords:** Notch, γ-secretase, apoptosis, cell cycle arrest

## Abstract

The present study was performed to explore the effects of Notch pathway inhibition on the proliferation and apoptosis of renal carcinoma cells. The expression levels of Notch1 and Jagged1 were examined by western blot analysis and immunohistochemistry in pathologically identified clear cell renal cell carcinoma (RCC) and normal kidney tissues. Next, γ-secretase inhibitor was used to suppress the Notch pathway in renal carcinoma cell lines. The proliferation was detected by 3-(4,5-dimethylthiazol-2-yl)-2,5-diphenyltetrazolium bromide assay and flow cytometry analysis was performed to determine the apoptosis, as well as cell cycle alteration. The expression of Notch1 and Jagged1 proteins was detected to be higher in tumor tissues than in non-neoplastic tissues by western blot analysis. The positive staining rates of Notch1 and Jagged1 in clear cell RCC were higher than in normal kidney tissues [95.3 vs. 36.4% (P<0. 05); 93.0 vs. 42.4% (P<0.05), respectively]. The expression levels of Notch1 and Jagged1 were found to statistically correlate with tumor size, grade, TNM stage and disease relapse. The suppression of the Notch pathway was associated with cell proliferation inhibition, as well as induced G2/M phase cell cycle arrest and cell apoptosis. The Notch pathway may be important in oncogenesis of clear cell RCC and the γ-secretase inhibitor may be a potential agent for target therapy of RCC.

## Introduction

Renal cell carcinoma (RCC) accounts for 2–3% of all adult malignant neoplasms. Annually, RCC affects ~150,000 individuals and causes ~78,000 mortalities worldwide (1). In addition, 25–30% of patients present with metastatic RCC at diagnosis (2). Although treatment with multikinase inhibitors has been shown to prolong progression-free survival rates, effective therapy for patients with metastatic advanced-stage RCC remains limited (3,4). Based on previous genetic and molecular studies, it has been postulated that additional tumorigenic events are required for the genesis of RCC, and investigations into these pathways may lead to the development of novel agents (5).

The Notch pathway is highly conserved and plays a crucial role in multiple cellular processes (6). Notch signaling is initiated through the interactions between the plasma-embedded Notch receptors (Notch1-4) and cell surface ligands (Jagged1 and Jagged2 and δ-like 1, 2 and 4) present on adjacent cells (6). This results in a conformational change in Notch to reveal the cleavage site 2 for metalloproteases (ADAM10 and ADAM17), which leaves a 12 amino acid stub of the Notch extracellular domain, required for subsequent recognition and cleavage by the γ-secretase complex. γ-secretase cleavage of Notch liberates the intracellular domain, which translocates to the nucleus, enabling gene transcription of Notch downstream targets (7).

Deregulated Notch signaling has been implicated in a number of tumor types, including hematological cancers and solid tumors (8–11). For RCC, it has been previously reported that the Notch signaling cascade is constitutively active in human RCC cell lines (12), and high expression of Notch has been associated with increased risk of metastasis (13). However, contrast theories remain that the expression of Notch receptors is downregulated and Notch signaling may function as a tumor suppressor in the progress of RCC (14).

Our previous study reported that Jagged1 is expressed at an elevated level in RCC and its overexpression may predict poor outcome in RCC patients (15). In order to further confirm the role of Notch singling in RCC, the present study detected the expression of Notch1 and Jagged1, as well as the effects of Notch pathway inhibition on the proliferation and apoptosis of renal carcinoma cells. The present study indicated that Notch plays a role in the tumorigenesis of RCC and highlights the potential use of γ-secretase inhibitor as a novel treatment for RCC.

## Materials and methods

### Reagents and antibodies

The antibodies used against Notch1 (polyclonal rabbit anti-human Notch1; ab27526) and Jagged1 (polyclonal goat anti-human Jagged1; sc-6011) were purchased from Abcam (Cambridge, UK) and Santa Cruz Biotechnology, Inc. (Santa Cruz, CA, USA), respectively. The peroxidase-conjugated mouse anti-goat IgG antibody was purchased from Shanghai Changdao Biologic Technology Co., Ltd. (Shanghai, China). Glyceraldehyde-3-phosphate dehydrogenase (GAPDH) was purchased from Santa Cruz Biotechnology, Inc. and the γ-secretase inhibitor, N-[N-(3,5-difluorophenacetyl)-l-alanyl]-S-phenylglycine t-butyl ester (DAPT), was purchased from Merck Biosciences (Darmstadt, Germany). Tissue culture media and fetal bovine serum (FBS) were purchased from Gibco (Fullerton, CA, USA). The Annexin V-fluorescein isothiocyanate (FITC) apoptosis detection kit was purchased from Beckman Coulter (Fullerton, CA, USA). Human renal carcinoma cell lines, 786-0, 769-p and Caki, were obtained from the Shanghai Institute of Cell Biology, Chinese Academy of Sciences (Shanghai, China).

### Patients and tissue samples

The present study was approved by the ethical committee of Zhongshan Hospital, Fudan University (no.2008-98; Shanghai, China). Each patient was involved after providing informed written consent. For western blot analysis, fresh tumor tissues (later verified as clear cell RCC) and normal (non-tumor) kidney tissues were obtained intraoperatively from eight patients who underwent radical nephrectomy at the Department of Urology, Zhongshan Hospital. The tissue samples were then snap-frozen in liquid nitrogen and stored at −80°C prior to analysis. For immunostaining, a total of 129 patients with pathologically verified clear cell RCC were enrolled consecutively. All patients underwent nephrectomy (partial or radical) performed at the Department of Urology, Zhongshan Hospital, between 2003 and 2008.

### Western blot analysis

The eight paired samples of RCC and normal renal tissues were solubilized in a lysis buffer (SDS) on ice. All lysates were centrifuged at 4°C at 10,000 × g for 10 min. The protein concentration was determined using the Bradford protein assay (Bio-Rad, Hercules, CA, USA). In total, 100 μg protein content from each sample was electrophoresed in 8% SDS-PAGE (Shanghai Changdao Biologic Technology Co., Ltd., Shanghai, China)and blotted on a nitrocellulose membrane (Bio-Rad). The membrane was blocked with 5% bovine serum albumin in 1× Tris-buffered saline (TBS) buffer at room temperature for 2 h and incubated with Notch1 (1:200) or Jagged1 (1:500) antibodies at 4°C overnight. Following three washes for 15 min in TBS, the membrane was incubated with the peroxidase-conjugated mouse anti-goat IgG antibody for 2 h at room temperature. Immunoreactive proteins were visualized by an enhanced chemiluminescence system (Immobilon, Millipore, Billerica, MA, USA) and GAPDH was used as the control for protein loading.

### Immunohistochemistry

Immunohistochemistry was performed using standard techniques with 129 cases of pathologically verified clear cell RCC. Briefly, 4-mm paraffin-embedded tissue sections were dewaxed in xylene and rehydrated in graded alcohols. Endogenous peroxidase was blocked using 3% hydrogen peroxide. Antigen retrieval was accomplished by boiling tissue sections in 10 mM citrate buffer (pH 6.0) for 10 min. Non-specific protein binding was performed by 30-min incubations with goat serum. These treatments were alternated with rinses in phosphate-buffered saline (PBS). The slides were then treated with Notch1 (1:200) or Jagged1 (1:100) antibodies for 1 h at room temperature. Next, the slides were rinsed with PBS and incubated with horse radish peroxidase-conjugated secondary antibody, followed by a rinse in PBS, incubation with 3,3′-diaminobenzidine staining and counterstaining with hematoxylin blue. Negative controls were performed by substituting the primary antibody with a non-immune serum. Control sections were treated in parallel with the samples.

### Evaluation of staining

All stained sections were evaluated by three independent investigators in a blind manner. The scoring was based on color intensity and extensity as previously described (16). Briefly, the proportion score was determined semi-quantitatively by assessing the whole tumor section at low magnification and each sample was scored on the following scale of 0–3: 0, no positive cells; 1, 1–20% of positive cells; 2, 21–60% of positive cells; and 3, 61–100% of positive cells. The intensity score was determined at high magnification as follows: 0, negative staining; 1, weakly positive staining; 2, moderately positive staining; and 3, markedly positive staining. Then, the total score of each section was calculated by sum of the two parameters.

### Cell culture

Human renal carcinoma cell lines, 786-0, 769-p and Caki, were cultured in Dulbecco’s modified Eagle’s medium (Invitrogen Life Technologies, Carlsbad, CA, USA) supplemented with 10% FBS, 2 mM L-glutamine, 100 U/ml penicillin and 0.1 mg/ml streptomycin in a humidified atmosphere with 5% CO_2_ incubator at 37°C. Cells were seeded in six-well plates at a density of 5×10^4^/well and allowed to adhere overnight. The medium was replaced with medium containing the inhibitor diluted in dimethyl sulphoxide (DMSO). For 3-(4,5-dimethylthiazol-2-yl)-2,5-diphenyltetrazolium bromide (MTT) and apoptosis detection, as well as cell circle analysis by flow cytometry, at least three independent experiments were performed.

### Cell viability assay

The antiproliferative effect of DAPT against various groups of cells was determined using the MTT (Sigma-Aldrich, St. Louis, MO, USA) assay. Briefly, cells were seeded in 96-well plates at a density of 1.0×10^4^ cells per well. Following overnight incubation, the cells were treated with DAPT (1, 2, 5, 10 and 20 μM) for 48 h. Following DAPT treatment, the medium was removed and 20 μl MTT (5 mg/ml in PBS) was added to each well. Following incubation for 4 h at 37°C, the supernatant was removed and the formazan crystals were solubilized by adding 150 μl DMSO. Viable cells were detected by measuring absorbance at 490 nm using MRX II absorbance reader (Dynex Technologies, Chantill, VA, USA). The reduction in viability of DAPT-treated cells was expressed as a percentage compared with non-DAPT-treated control cells. Control cells were considered to be 100% viable.

### Detection of apoptotic cells by flow cytometry

Cells were plated in six-well plates (2 ml/well) at a density of 5×10^5^ cells/ml and incubated overnight. DAPT of various concentrations (1, 2, 5 and 10 μM) was then added into each well and incubated for 48 h. The cells were collected and washed with PBS, followed by resuspension in binding buffer at a concentration of 1×10^6^ cells/ml. A total of 100 ml (1×10^5^ cells) of the solution was removed and mixed with Annexin V-FITC and propidium iodide (PI) according to the manufacturer’s instructions. The mixed solution was incubated in the dark at room temperature for 15 min, 400 μl dilution buffer was then added to each tube and cell apoptosis analysis was performed using the Beckman Coulter FC500 Flow Cytometry system (Beckman Coulter) within 1 h.

### Analysis of cell cycle distribution

Cell cycle analysis was performed using the Coulter DNA Prep™ Reagents kit (Beckman Coulter). Cells were prepared as previously described. The cells were then exposed to various concentrations of DAPT (1, 2, 5 and 10 μM) for 48 h at 37°C. Cells were harvested, washed with cold PBS, fixed with 70% ethanol and stored at 4°C for subsequent cell cycle analysis. For detecting DNA content, cells were incubated in the dark at room temperature with 0.5 ml RNase A for 20 min and with 1 ml PI for 20 min. The DNA content of the cells was measured using the Beckman Coulter FC500 Flow Cytometry system. The percentage of cells in G1, S and G2/M phases was calculated.

### Statistical analysis

Difference of immunostaining between neoplastic and normal kidney tissues was detected by the χ^2^ test, as well as the correlation between protein expression and clinical and pathological characteristics. Statistical analyses were performed using a statistical software package (SPSS, version 16.0; SPSS, Inc., Chicago, IL, USA). P<0.05 was considered to indicate a statistically significant difference and all P-values were two-sided.

## Results

### Western blot analysis

**Figure 1:**
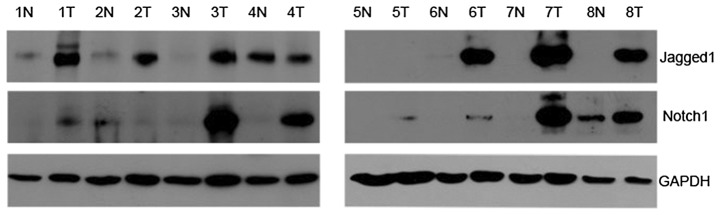
Expression of Notch1 and Jagged1 by western blot analysis. N, non-neoplastic renal tissue; T, renal cell carcinoma tissue.

As Fig. 1 shows, the protein of Notch1 was expressed in adjacent non-neoplastic tissues and RCC tissues, with specific bands at 80 kDa. Notch1 was found to be upregulated in seven cases of RCC tissues (7/8; 87.5%) compared with paired non-neoplastic tissues. Similarly, Jagged1 was detected at 150 kDa. The expression of Jagged1 was higher in six tumor tissues (6/8, 75.0%) than in paired non-neoplastic tissues.

### Clinical and pathological characteristics

In total, 129 cases with clear cell RCC were enrolled for immunostaining of Jagged1. In total, eight cases were collected for western blot analysis. The clinical and pathological characteristics of the patients are listed in Table I.

### Immunohistochemistry

**Figure 2:**
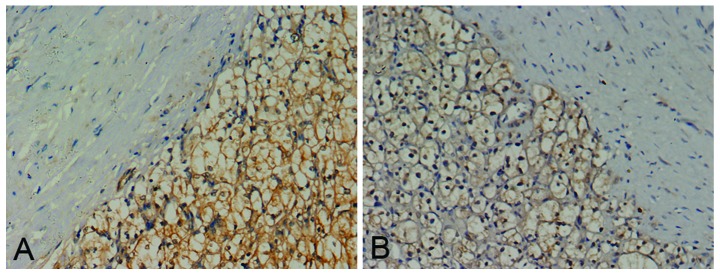
Expression of (A) Notch1 and (B) Jagged1 by immunohistochemistry (magnification, ×200).

Notch1 and Jagged1 staining was present mainly in the cell membrane and/or cytoplasm (Fig. 2). The positive staining rate of Notch1 in RCC tissues was 95.3% (123/129), compared with 36.4% (12/33) in normal kidney tissues (P<0.05; χ^2^=65.8). The positive staining rate of Jagged1 in RCC tissues was 93.0% (120/129), while that in normal kidney tissues was 42.4% (14/33) (P<0.05; χ^2^=47.1). Notch1 and Jagged1 exhibited a significantly higher expression in RCC tissues than in normal kidney tissues.

Low expression was designated as a total score of 0–3, while high expression was designated as a total score of 4–6. Tumors were subdivided according to protein expression level into various groups. The expression level of Notch1 was found to statistically correlate with nuclear grade (P=0.025), TNM stage (P=0.037) and tumor size (P=0.002). The expression level of Jagged1 was also found to statistically correlate with nuclear grade (P=0.001), TNM stage (P=0.002) and tumor size (P=0.016), which has been mentioned in our previous study (15). In particular, cases with higher Notch1 or Jagged1 expression showed higher rates of disease relapse, with P=0.024 and P<0.001, respectively (Tables II and III).

### DAPT inhibits renal carcinoma cell growth

**Figure 3:**
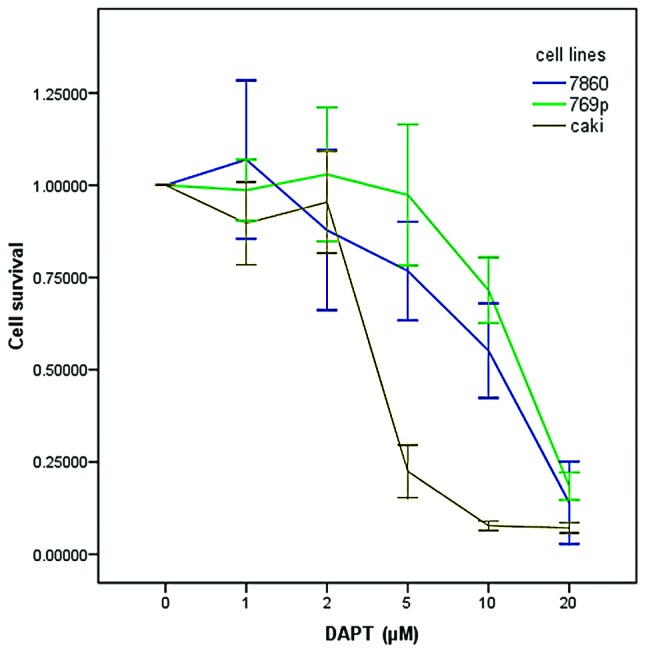
DAPT-inhibited cell viability of human renal carcinoma cell lines. Viability of cells was determined by the 3-(4,5-dimethylthiazol-2-yl)-2,5-diphenyltetrazolium bromide assay. DAPT, N-[N-(3,5-difluorophenacetyl)-l-alanyl]-S-phenylglycine t-butyl ester.

In order to investigate the potential effects of DAPT on the growth and viability of human renal carcinoma cells, various cell lines (786-0, 769-p and Caki) were treated with DAPT at various concentrations (1, 2, 5, 10 and 20 μM) by MTT assay. As shown in Fig. 3, inhibition of cell proliferation by DAPT was generally in a dose-dependent manner. The IC_50_ dose of DAPT for the proliferation of renal carcinoma cells was ~12.8, 11.4 and 4.9 μM for 786-0, 769-p and Caki renal carcinoma cell lines, respectively.

### DAPT induces G2/M phase cell cycle arrest

**Figure 4:**
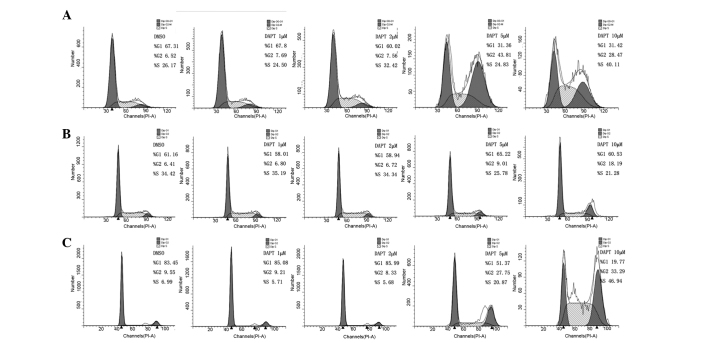
DAPT-induced G2/M phase cell cycle arrest in human renal carcinoma cell lines. (A) 786-0, (B) 769-p and (C) Caki cells were treated with various concentrations of DAPT for 48 h and analyzed by flow cytometry. DAPT, N-[N-(3,5-difluorophenacetyl)-l-alanyl]-S-phenylglycine t-butyl ester; DMSO, dimethyl sulphoxide.

Based on the growth inhibitory response of DAPT treatment in cells, its effect on cell cycle distribution was next examined. Renal carcinoma cells were treated with various concentrations of DAPT for 48 h and analyzed by flow cytometry. As shown in Fig. 4, the level of G2/M-phase arrest was observed. Following treatment with 1, 2, 5 and 10 μM DAPT for 48 h, the rate of G2/M phase for 786-0 cells was increased by 7.69, 7.56, 43.81 and 28.47%, respectively. While for 769-p cells, the rate was 6.80, 6.72, 9.01 and 18.19%, respectively, and for Caki cells, the rate was 9.21, 8.33, 27.75 and 33.29%, respectively. These results suggested that DAPT induces G2/M phase cell cycle arrest in renal carcinoma cells.

### DAPT induces apoptosis in renal carcinoma cells

**Figure 5:**
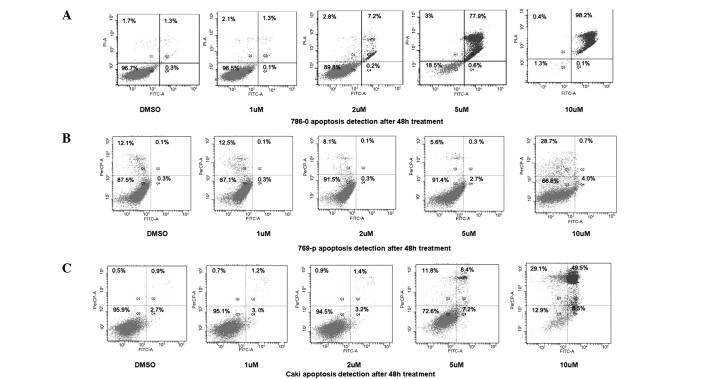
DAPT treatment-induced dose-dependent apoptosis in (A) 786-0, (B) 769-p and (C) Caki human renal carcinoma cell lines using a double-staining method with Annexin V-FITC/PI. DAPT, N-[N-(3,5-difluorophenacetyl)-l-alanyl]-S-phenylglycine t-butyl ester; FITC, fluorescein isothiocyanate; PI, propidium iodide; DMSO, dimethyl sulphoxide.

To determine whether the DAPT-induced growth inhibition was mediated by apoptosis, flow cytometry was further used to identify the cell death types. As shown in Fig. 5, 786-0, 769-p and Caki RCC cell lines treated with DAPT showed a dose-dependent increase in the levels of apoptosis.

## Discussion

The Notch pathway is critical in the determination of cell fates by regulating cell growth, differentiation and apoptosis (6). It plays an oncogenic or a tumor suppressive role, depending on the cancer type, the other signaling pathways involved and activation of the Notch receptor (17).

Previously, the aberrant regulation of Notch signaling has been implicated in tumorigenesis; however, conflicting theories concerning the role of the Notch pathway in RCC exist (12,14,15). In the current study, the expression of Notch1 and Jagged1 was detected and an elevated level was shown in neoplastic tissue as compared with that in normal kidney tissue, which was also confirmed by western blot analysis. In addition, the expression levels of Notch1 and Jagged1 were found to markedly correlate with tumor size, grade and TNM stage, as well as disease relapse, suggesting that the Notch pathway may be associated with the oncogenesis process of RCC.

When the γ-secretase inhibitor (DAPT) was applied to renal carcinoma cell lines, the proliferation was decreased. The suppression by DAPT was associated with induced G2/M-phase cell cycle arrest, as well as cell apoptosis. The present study indicated the oncogenic role of Notch signaling in the development of RCC. Notably, the 769-p cells appeared to be less sensitive to γ-secretase treatment than the other two cell lines. The mechanism by which these cells partially escaped inhibition of γ-secretase cleavage remains to be determined. It must be noted that specific T-cell acute lymphoblastic leukemia (T-ALL) cells harboring Notch1 activating mutations were refractory to γ-secretase treatment (18). It is important to clarify whether mutations in the Notch pathway are present in the subset of RCC.

The mechanism involved with the oncogenic role of Notch may be multiple. Firstly, several previous studies have shown that Notch signaling is pivotal for tumor angiogenesis (10,19,20). Secondly, the regulatory effect of Notch signaling has been reported to be associated with the suppression of p21Cip1 and p27Kip1, two cyclin-dependent kinase inhibitory proteins of pivotal importance in cell cycle control (12). This result is consistent with the results of the present study that inhibition of the Notch pathway leads to considerable inhibition of cell cycle progression. Finally, according to our previous study, the phosphatidylinositide 3-kinases (PI3K)/protein kinase B (Akt) pathway is regulated by Notch1 activation and elevated Notch1 signaling activity may exert its growth-promoting effects via the PI3K/Akt pathway (21).

γ-secretase is a protease complex and is composed of a catalytic subunit (presenilin-1 or −2) and accessory subunits (presenilin enhancer 2, anterior pharynx-defective 1 and nicastrin) (22). Since γ-secretase inhibitors are able to prevent Notch receptor activation, the γ-secretase complex may be a potential therapeutic target in a wide array of carcinomas. Inhibition of Notch signaling by a γ-secretase inhibitor, PF-03084014, resulted in suppression of tumor cell proliferation and induction of apoptosis in T-ALL (23). In breast cancer, Rasul *et al* showed that inhibition of γ-secretase activity in breast cancer cell lines induced G2/M arrest and downregulated antiapoptotic proteins leading to cell death (24).

Although, for clear cell RCC, several kinase inhibitors, including sorafenib and sunitinib, show effects on the progression-free survival rate for specific patients. However, the side effects of kinase inhibitors must not be underestimated (25). The efficacy of these drugs is likely to be associated with their capacity to inhibit hypoxia-inducible factor-mediated autocrine growth factor signaling and proangiogenic effects. Notably, loss of von Hippel-Lindau is associated with good prognosis in clear cell RCC (26,27). The therapeutic effect of γ-secretase inhibition on clear cell RCC tumor growth indicates that the inhibition of Notch signaling presents at least a complementary therapeutic approach for treatment of clear cell RCC. In the present study, inhibition of clear cell RCC cells with DAPT led to a considerable decrease of cell proliferation and increased apoptosis. The results support the therapeutic effect of DAPT for clear cell RCC. Considering the limitation of kinase inhibitors, a comprehensive evaluation of the optimal administration regime of γ-secretase inhibitors is of priority for clear cell RCC.

Deficiencies remain in the current study. Firstly, γ-secretase inhibitor blocked the Notch pathway without specifying the individual contributions of the respective receptors or ligands. We supposed that targeting each receptor or ligand using siRNA is necessary to elucidate their respective contribution to proliferation. Secondly, since tumorigenesis is a complicated and comprehensive pathway, the underlying detailed mechanism of this difference also requires further study.

In conclusion, the current study indicated that Notch signaling is important in the tumorigenesis of RCC. The γ-secretase inhibitor (DAPT) has the potential of being a novel therapeutic regimen towards RCC, although, further investigation is required.

## Figures and Tables

**Table I:** Clinicopathological characteristics of clear cell RCC cases.

Characteristics	IHC (n=129)	Western blotting (n=8)
Gender, n (%)		
Male	84 (65.1)	6 (75.0)
Female	45 (34.9)	2 (25.0)
Age, years		
Mean	55.5	61.5
Range	27–83	55–76
Surgery, n (%)		
Radical nephrectomy	122 (94.6)	7 (87.5)
Partial nephrectomy	7 (5.4)	1 (12.5)
Tumor size, cm		
Mean	5.3	4.3
Range	1.5–15	2.5–12
TNM stage, n (%)		
I	81 (62.8)	2 (25.0)
II	17 (13.2)	3 (37.5)
III	24 (18.6)	2 (25.0)
IV	7 (5.4)	1 (12.5)
Fuhrman grade, n (%)		
1	53 (41.1)	3 (37.5)
2	54 (41.9)	3 (37.5)
3	18 (14)	1 (12.5)
4	4 (3.0)	1 (12.5)
Relapse, n (%)		
Yes	37 (28.7)	1 (12.5)
No	92 (71.3)	7 (87.5)

RCC, renal cell carcinoma; IHC, immunohistochemistry.

**Table II:** Correlation between Notch1 and clinicopathological characteristics.

	Age, years		Gender		Tumor size, cm		TNM stage		Fuhrman grade		Relapse	
						
Notch1	<55	≥55	Male	Female	<5.0	≥5.0	I+II	III+IV	1+2	3+4	Yes	No
Low expression, n	37	32	44	25	41	28	57	12	62	7	14	55
High expression, n	29	31	40	20	19	41	40	20	45	15	23	37
χ^2^	0.359		0.119		9.936		4.373		5.006		5.108	
P-value	0.549		0.730		0.002		0.037		0.025		0.024	

**Table III:** Correlation between Jagged1 and clinicopathological characteristics.

	Age, years		Gender		Tumor size, cm		TNM stage		Fuhrman grade		Relapse	
						
Jagged1	<55	≥55	Male	Female	<5.0	≥5.0	I+II	III+IV	1+2	3+4	Yes	No
Low expression, n	40	27	46	21	38	29	58	9	63	4	9	58
High expression, n	26	36	38	24	22	40	39	23	44	18	28	34
χ^2^	3.867		0.769		5.835		9.667		12.107		15.848	
P-value	0.053		0.380		0.016		0.002		0.001		<0.001	

## References

[1] Zbar B, Klausner R, Linehan WM (2003). Studying cancer families to identify kidney cancer genes. Annu Rev Med.

[2] Ljungberg B, Hanbury DC, Kuczyk MA (2007). Renal cell carcinoma guideline. Eur Urol.

[3] Escudier B, Eisen T, Stadler WM (2007). Sorafenib in advanced clear-cell renal-cell carcinoma. N Engl J Med.

[4] Motzer RJ, Hutson TE, Tomczak P (2007). Sunitinib versus interferon alfa in metastatic renal-cell carcinoma. N Engl J Med.

[5] Kim WY, Kaelin WG (2004). Role of VHL gene mutation in human cancer. J Clin Oncol.

[6] Artavanis-Tsakonas S, Rand MD, Lake RJ (1999). Notch signaling: cell fate control and signal integration in development. Science.

[7] Bettenhausen B, Hrabĕ de Angelis M, Simon D, Guenet JL, Gossler A (1995). Transient and restricted expression during mouse embryogenesis of Dll1, a murine gene closely related to Drosophila Delta. Development.

[8] Ellisen LW, Bird J, West DC (1991). TAN-1, the human homolog of the Drosophila notch gene, is broken by chromosomal translocations in T lymphoblastic neoplasms. Cell.

[9] Miele L, Golde T, Osborne B (2006). Notch signaling in cancer. Curr Mol Med.

[10] Qi R, An H, Yu Y (2003). Notch1 signaling inhibits growth of human hepatocellular carcinoma through induction of cell cycle arrest and apoptosis. Cancer Res.

[11] Shi W, Harris AL (2006). Notch signaling in breast cancer and tumor angiogenesis: cross-talk and therapeutic potentials. J Mammary Gland Biol Neoplasia.

[12] Sjölund J, Johansson M, Manna S (2008). Suppression of renal cell carcinoma growth by inhibition of Notch signaling in vitro and in vivo. J Clin Invest.

[13] Ai Q, Ma X, Huang Q (2012). High-level expression of Notch1 increased the risk of metastasis in T1 stage clear cell renal cell carcinoma. PLoS One.

[14] Sun S, Du R, Gao J (2009). Expression and clinical significance of Notch receptors in human renal cell carcinoma. Pathology.

[15] Wu K, Xu L, Zhang L, Lin Z, Hou J (2010). High Jagged1 expression predicts poor outcome in clear cell renal cell carcinoma. Jpn J Clin Oncol.

[16] Massi D, Tarantini F, Franchi A (2006). Evidence for differential expression of Notch receptors and their ligands in melanocytic nevi and cutaneous malignant melanoma. Mod Pathol.

[17] Weng AP, Aster JC (2004). Multiple niches for Notch in cancer: context is everything. Curr Opin Genet Dev.

[18] Weng AP, Ferrando AA, Lee W (2004). Activating mutations of NOTCH1 in human T cell acute lymphoblastic leukemia. Science.

[19] Ridgway J, Zhang G, Wu Y (2006). Inhibition of Dll4 signalling inhibits tumour growth by deregulating angiogenesis. Nature.

[20] Zeng Q, Li S, Chepeha DB (2005). Crosstalk between tumor and endothelial cells promotes tumor angiogenesis by MAPK activation of Notch signaling. Cancer Cell.

[21] Xu L, Zhu Y, Xu J (2012). Notch1 activation promotes renal cell carcinoma growth via PI3K/Akt signaling. Cancer Sci.

[22] Lazarov VK, Fraering PC, Ye W, Wolfe MS, Selkoe DJ, Li H (2006). Electron microscopic structure of purified, active gamma-secretase reveals an aqueous intramembrane chamber and two pores. Proc Natl Acad Sci USA.

[23] Wei P, Walls M, Qiu M (2010). Evaluation of selective gamma-secretase inhibitor PF-03084014 for its antitumor efficacy and gastrointestinal safety to guide optimal clinical trial design. Mol Cancer Ther.

[24] Rasul S, Balasubramanian R, Filipović A, Slade MJ, Yagüe E, Coombes RC (2009). Inhibition of gamma-secretase induces G2/M arrest and triggers apoptosis in breast cancer cells. Br J Cancer.

[25] Park SJ, Lee JL, Park I (2012). Comparative efficacy of sunitinib versus sorafenib as first-line treatment for patients with metastatic renal cell carcinoma. Chemotherapy.

[26] Banks RE, Tirukonda P, Taylor C (2006). Genetic and epigenetic analysis of von Hippel-Lindau (VHL) gene alterations and relationship with clinical variables in sporadic renal cancer. Cancer Res.

[27] Yao M, Yoshida M, Kishida T (2002). VHL tumor suppressor gene alterations associated with good prognosis in sporadic clear-cell renal carcinoma. J Natl Cancer Inst.

